# Evaluation of Lipid Oxidation, Volatile Compounds and Vibrational Spectroscopy of Silver Carp (*Hypophthalmichthys molitrix*) during Ice Storage as Related to the Quality of Its Washed Mince

**DOI:** 10.3390/foods10030495

**Published:** 2021-02-25

**Authors:** Sasinee Kunyaboon, Kanjana Thumanu, Jae W. Park, Chompoonuch Khongla, Jirawat Yongsawatdigul

**Affiliations:** 1 School of Food Technology, Institute of Agricultural Technology, Suranaree University of Technology, Nakhon Ratchasima 30000, Thailand; sasinee2011@gmail.com; 2 Synchrotron Light Research Institute (Public Organization), Nakhon Ratchasima 30000, Thailand; kanjanat@slri.or.th; 3 Seafood Lab and Department of Food Science and Technology, Oregon State University, 2001 Marine Drive #253, Astoria, OR 97103, USA; jae.park@oregonstate.edu; 4 Department of Applied Biology, Faculty of Sciences and Liberal Arts, Rajamangala University of Technology Isan, Nakhon Ratchasima 30000, Thailand; chompoonuch.2840@gmail.com

**Keywords:** silver carp, washed mince, FTIR, FT-Raman, lipid oxidation, volatile compounds

## Abstract

Changes in the lipid oxidation of silver carp (*Hypophthalmichthys molitrix*) stored in ice for 14 days and that of its respective washed mince were evaluated. Total lipid, phospholipid, polyunsaturated fatty acid (PUFA) and monounsaturated fatty acid (MUFA) contents of the skin, belly flap and mince decreased as the storage time in ice increased. The washing process decreased the lipid contents but concentrated their phospholipid counterparts. The fish belly flap exhibited the highest thio-barbituric acid reactive substances (TBARS) value, while the mince had the lowest. 1-Hexanol, 1-octen-3-ol, and 1-hexanal were key volatile compounds detected in the belly flaps of fish stored for 7–14 days. Hexanal was the only major volatile compound found in washed mince prepared from fish stored for an extended period in ice, but in a much lower amount compared with that in the belly flap. FTIR (Fourier transform infra-red) spectra revealed a decrease in the number of *cis* double bonds, methylene groups and phosphate groups in lipids extracted from fish stored in ice for 7–14 days as compared with those extracted from fresh fish. Principle component analysis (PCA) revealed that the FT-Raman band at 1747 cm^−1^ could be a potential marker for tracking the degree of lipid oxidation in the belly flap of silver carp stored in ice. In addition, IR bands indicating phosphate group (925, 825 cm^−1^) in oil extracted from washed mince were correlated with the extent of the lipid oxidation of the raw material.

## 1. Introduction

Silver carp (*Hypophthalmichthys molitrix*) is an important freshwater fish species with global production of 4,704,673 tons in 2017 [1]. At the industry level, silver carp has proven useful as a potential raw material for surimi production, providing good gel-forming ability with exceptional white color [2,3]. In tropical surimi production, whole fish are typically kept in ice before being processed. It is well recognized that various parts of the fish body contain different lipid contents, leading to varied degree of lipid oxidation during ice storage. Lipid oxidation is known to produce undesirable flavors and oxidized products that induce protein oxidation. This ultimately leads to a deterioration in the texture and sensory characteristics of surimi. Fish freshness quality has been extensively studied with regard to nucleotide degradation and changes in protein conformation. However, changes in the lipid oxidation of silver carp during ice storage have not been well characterized.

Many methods have been applied to evaluate lipid oxidation in fish and surimi, including (1) measuring changes in oxidative substrates, such as fatty acids, total lipid and phospholipid contents; and (2) assessing the quantity of primary and secondary products of oxidation. The thio-barbituric acid reactive substances (TBARS) level, which is a widely used lipid oxidation indicator, has some limitations. Malondialdehyde (MDA) is only one of many possible secondary oxidation products formed [4]. In addition, other compounds that do not result from the oxidation process can contribute to TBARS levels [5]. Recently, the evaluation of volatile compounds has become an additional indicator of lipid oxidation in fish and surimi samples [6]. In addition, Fourier transform infrared (FT-IR) and FT-Raman spectroscopy are techniques that can be used to monitor oxidative changes. FT-Raman spectroscopy was used to monitor lipid oxidation in hake fillets during frozen storage [7] and in beef during repeated freeze-thaw cycles [8]. Vibrational spectroscopy can provide additional insightful information at the molecular level. Therefore, the objective of this study was to evaluate the lipid oxidation of various parts of silver carp stored in ice for 14 days. In addition, the lipid oxidation of washed mince prepared from fish stored in ice for various periods was analyzed. Changes in volatile compounds were also investigated. Vibrational spectroscopic techniques, namely FTIR and FT-Raman, were also applied to monitor the lipid oxidation of oil extracted from raw materials stored in ice for up to 14 days.

## 2. Materials and Methods

### 2.1. Sample Preparation

Live silver carp (*Hypophthalmichthys molitrix*) weighing 1.0–1.5 kg were transported from the Khon Kaen Inland Fisheries Research and Development Center to a laboratory at Suranaree University of Technology within 4 h. Upon arrival, fish were stunned by an accurate blow to the head, with regard to animal welfare law, and immediately packed in polystyrene foam boxes filled with ice, with a fish-to-ice ratio of approximately 1:2. The polystyrene foam boxes were kept in a cold room (4 °C) for 14 days. Ice was added every 2 days. At 0, 7 and 14 days of storage, fish were randomly selected and washed with tap water (27 °C). Fish skin and belly flap were manually separated and collected. Fish flesh was minced using a grinder with a 5-mm perforation plate. Fish mince was washed three times with potable water (<5 °C) at a mince/water ratio of 1:3. The third washing cycles were carried out using the same volume of 0.3% NaCl solution. Centrifugation was carried out at 5000× *g* for 15 min at 4 °C at each washing step. Any floating matter, including muscle tissue and fats, was manually removed after centrifugation. Fish skin, belly flap and unwashed and washed mince in a total of 36 samples were vacuum packed and kept at −80 °C and used within 1 week.

### 2.2. Analysis of Lipid and Fatty Acids

Total lipid content was analyzed according to the Folch method [9] with slight modifications. Each ground sample (30 g) was homogenized with 180 mL of a chloroform and methanol solution (2:1) for 1 min and centrifuged at 2000× *g*, 4 °C, for 10 min. The solution was then filtered through Whatman No. 1 filter paper into a separatory funnel. Chloroform (60 mL), deionized water (60 mL) and 0.58% NaCl (10 mL) were then added and thoroughly mixed. After phase separation, the lower layer of the chloroform phase was collected, and anhydrous sodium sulfate was added to remove water. The chloroform phase was then filtered through Whatman No.1 filter paper. The chloroform was evaporated under nitrogen. The extracted lipid was determined gravimetrically and total lipid was expressed as g/100 g dry weight. Some portions of the extracted oil were kept at −80 °C before further analysis of phospholipid, fatty acid profile and FTIR and FT-Raman spectra.

Phospholipid content was estimated as described by Eymard et al. [10]. Extracted lipid was dissolved in chloroform (0.25 mg/mL). Then, 2 mL of solution was mixed with 1 mL of thiocyanate reagent (0.10 M ferric chloride hexahydrate and 0.40 M ammonium thiocyanate). The mixture was centrifuged at 750× *g*, 4 °C, for 10 min. The red lower layer was collected for absorbance measurement at 488 nm. Phosphatidylcholine (PC) at various concentrations, ranging from 0 to 0.1 mg/mL, was used as a standard. Phospholipid content was expressed as g PC/100 g dry sample. 

Fatty acid composition and quantification was evaluated using gas chromatography (GC) according to the Association of Official Analytical Chemists (AOAC) method [11]. Methylation of fatty acid was performed as follows. Extracted lipid (25 mg) was weighted in a 10-mL screw cap tube, and 1.5 mL of 0.5 M NaOH in methanol was added. The mixture was flushed with nitrogen gas for 30 s and heated at 85 °C for 2 min. Subsequently, 1 mL of internal standard (C17 fatty acid) and 2 mL 14% boron trifluoride (BF_3_) in methanol was added. The mixture was flushed with nitrogen gas and reheated again at 85 °C for 30 min and mixed with 1 mL of isooctane. Subsequently, 5 mL of saturated NaCl solution was added to separate isooctane phase from the methanol and water phase. The mixture was reextracted with isooctane, and the isooctane phase was collected until 5 mL of the extraction was obtained. The isooctane phase containing fatty acid methyl esters (FAME) was filtered through a 0.45 µm syringe filter before GC analysis. 

GC (7890A, Agilent technologies, Santa Clara, CA, USA) equipped with a flame ionization detector (FID) and an SP2560 capillary column (100 m × 0.20 µm film thickness × 0.25 mm internal diameter, Supelco Co., Ltd., Bellefonete, PA, USA) was used for FAME analysis. The carrier gas was helium with a flow rate of 1.0 mL/min. The temperature of the injection port and detector were maintained at 250 °C. Identification and quantification of fatty acids were performed using external standards (Supelco 37 FAME, Sigma–Aldrich Co., St.Louis, MO, USA) at concentrations ranging from 0 to 10 mg/mL and was expressed as mg/g dry sample.

### 2.3. Heme Iron Content

Heme iron content was determined according to the method of Clark et al. [12]. Two grams of ground sample were added to 20 mL of acid–acetone mixture (40 mL of acetone, 9 mL of water and 1 mL of concentrated hydrochloric acid). The mixture was homogenized at 10,000 rpm for 30 s. Then, 20 mL of the acid-acetone mixture was added again, and the mixture was kept in the dark for 1 h. The mixture was centrifuged at 2200× *g* for 10 min. The supernatant was collected and filtered through Whatman No.1, and the absorbance was measured at 640 nm. The concentration of total pigments in the sample (μg hematin/g sample) was calculated by multiplying the absorbance by a factor of 6800 and then dividing by the sample weight. The iron content was calculated using a factor of 0.0882 μg iron/μg hematin. The heme iron content was expressed as mg/100 g sample [13].

### 2.4. Thiobarbituric Acid Reactive Substances (TBARS) 

TBARS values were determined according to Reitznerová [4]. Two grams of sample were homogenized with 7.5% trichloroacetic acid (TCA) for 30 s and centrifuged at 10,000× *g*, 4 °C, for 10 min. The homogenate was filtered through Whatman no. 1 filter paper. The supernatant (2 mL) was mixed with 2 mL of 0.02 M TBA solution. The sample was heated at 95 °C for 20 min and cooled in ice or at room temperature for 10 min. The absorbance was measured at 532 nm. 1,1,3,3-Tetraethyloxypropane (TEP) was used as a standard. The TBARS value was expressed as ng of malonaldehyde/kg dry sample.

### 2.5. Determination of Volatile Compounds

Volatile compounds were detected by head space solid-phase microextraction gas chromatography-mass spectrometry (SPME/GC-MS). The SPME fiber was coated with carboxen–divinylbenzene–polydimethylsiloxane (CAR/DVB/PDMS) (Supelco, Bellefonte, PA, USA). One gram of ground sample was placed in a 20 mL round bottom vial and then mixed with 3 mL of deionized water, 0.7 g of NaCl, 10 µL of 7.2% butylated hydroxytoluene (BHT) in 70% ethanol and 30 µL of 100 ppm cyclohexanol as an internal standard. The vial was sealed with polytetrafluoroethylene (PTFE)/silicone septa (Agilent, Santa Clara, CA, USA). The mixture was equilibrated at 60 °C for 10 min. Volatile compounds were then analyzed on a 450-GC coupled to a 320-MS Quadrupole mass spectrometer (Bruker Daltonics, Billerica, MA, USA). The GC oven temperature program started at 3 °C for 5 min, followed by heating at 3 °C/min to 70 °C, then heating at 10 °C/min to 200 °C, and then heating at 20 °C/min to 260 °C, and this temperature was maintained for 5 min. Helium was employed as the carrier gas in the linear flow control mode with a constant column flow of 1.0 mL/min. The quadrupole mass spectrometer was operated in the electron impact (EI) mode, and the source temperature was set at 70 eV and 200 °C. Volatile compounds were identified by searching MS library and using Kovats indices (RI). 

### 2.6. Vibrational Spectroscopy 

FT-Raman spectroscopy was performed on a Bruker RAM II FT-Raman module coupled to a Bruker Vertex 70v interferometer (Bruker Co., Ettlingen, Germany). The excitation source was an Nd:YAG laser at 1064 nm with 500 mW of laser power. The scattered radiation was collected from the range between 4000 and 400 cm^−1^ with a resolution of 4 cm^−1^ and 256 scans. Extracted lipid samples were placed in a stainless steel cup inserted in a sample holder and monitored via video camera. A Ge detector used liquid nitrogen as the coolant. Instrument control and spectral acquisition were performed using OPUS 7.2 (Bruker Optics Ltd., Ettlingen, Germany). At least 12 Raman spectra per sample were collected, averaged and normalized using the OPUS program, version 7.2. The integrated intensity of the second derivative (13-point smoothing) was then computed to distinguish the overlapped peaks. The result was expressed as the relative integrated intensity. FT-IR spectroscopy with a single reflection attenuated total reflectance (ATR) sampling module, coupled with MCT detector and cooled with liquid nitrogen (Bruker Tensor 27, Bruker Optics Ltd., Ettlingen, Germany), was also used to collected IR spectra. Extracted lipid (20 µL) was placed in contact with a horizontal ATR plate. IR spectra were obtained from an interval of 4000–600 cm^−1^ at a 4 cm^−1^ spectral resolution with 64 scans. At least 30 spectra were collected from each sample, and they were analyzed with the OPUS 7.2 program. Spectra were taken from three replications and then averaged. Normalization and the second derivative were carried out. The results were expressed as the relative integrated intensity.

### 2.7. Statistical Analyses

Statistical analyses were performed using SPSS 17.0 software (SPSS Inc, Chicago, IL, USA). Statistical evaluation was conducted using one-way analysis of variance (ANOVA). Comparison of means within each tissue at various storage time was carried out by Duncan’s new multiple range tests. The significance of difference was defined at 95% confidence interval (*p* < 0.05). Principal component analysis (PCA) of all measured parameters were performed on means results using the XLSTAT software (Addinsoft, New York, NY, USA).

## 3. Results and Discussion

### 3.1. Changes in Lipids

The highest lipid content was found in the belly flap (33.9–40.6%), followed by muscle (9.0–11.5%), and skin (7.0–8.8%) (Table 1). Lipids in fish are typically located in subcutaneous tissue, belly flap, muscle, mesentery, liver and head [14]. Moradi et al. [15] reviewed that the lipid content in the skin of lean fish ranged from 0.2–3.9% (wet basis), while that of fatty fish could be higher than 50% (wet basis). In contrast, lipid content in fish muscle ranged from <2% in lean fish to >8% in fatty fish [15], which was equivalent to 10% and 40% (dry basis), respectively. Thilakarathne and Attygalle [16] reported that the highest lipid content (6.52% wet basis) in Indo-Pacific sailfish (*Istiophorus platypterus*) was in the skin, followed by the belly flap, which contained 3.91%. Distribution of lipids in fish body appeared to vary with species. The study demonstrated that lipids are primarily located at the belly flap in silver carp. Among various parts of the studied raw material, muscle tissues contained the highest phospholipid content, which is an important component of membranes. 

The lowest phospholipid content was found in the skin, which is composed of subcutaneous tissues that mainly contain fat cells composed of triacylglycerols. Aursand et al. [17] reported lower contents of phospholipid in belly flap and higher proportion of neutral lipid. When washing was performed, 35–45% of lipids were removed (Table 1). Tongnuanchan et al. [18] reported that the lipid content of washed red tilapia mince decreased by 14.4%, in comparison with that found in unwashed mince. The removed fat was mainly triacylglycerols, which were clearly separated and appeared as floating fats after centrifugation. However, the phospholipid content of washed mince was increased when compared with unwashed mince, regardless of storage time (Table 1). Membrane lipids bind to membrane proteins, making it difficult to remove them by washing [19]. Myo-fibillar proteins remained in the washed mince along with membrane lipids. Eymard et al. [10] also reported that washing horse mackerel led to a greater reduction in neutral lipids as compared with polar lipids.

Total lipid content of all tissues from raw materials decreased during ice storage (*p* < 0.5, Table 1), but those of washed mince were comparable (*p* > 0.05). A decrease in lipid content during ice storage was likely to be due to the degradation of lipid by endogenous lipases and/or lipid oxidation. Chaijan et al. [20] reported that, during ice storage, triacyl-glycerols in sardine (*Sardinella gibbosa*) muscle decreased, while free fatty acid, diglycerol and mono-glycerol contents increased. This suggested that triacyl-glycerols were hydrolysed into free fatty acids. The phospholipid content of all samples tended to decrease with storage time, particularly in the muscle (*p* < 0.05, Table 1). This indicated oxidation of membrane lipids during ice storage. 

The belly flap contained the highest saturated fatty acid (SFA), monounsaturated fatty acid (MUFA) and polyunsaturated fatty acid (PUFA) contents, while washed mince contained the lowest (*p* < 0.05, Table 2). These results are in agreement with the total lipid content (Table 1). The predominant SFA and MUFA in all samples were palmitic acid (C16:0) and oleic acid (C18:1n9c). The major polyunsaturated fatty acid (PUFA) in the skin and belly flap was linolenic acid (C18:3n3). The main PUFA in unwashed and washed mince was docosahexaenoic acid (DHA, C22:6n3). These results suggested that unwashed and washed mince from silver carp contained PUFA, which is prone to lipid oxidation. MUFA and PUFA contents of all samples decreased with a concomitant increase in SFA after raw materials were stored in ice for 14 days. The reduction of MUFA and PUFA was probably due to lipid oxidation during ice storage. The increase in SFA was likely to be due to the degradation of MUFA and PUFA, which, in turn, increased the proportion of SFA [21]. These results are in agreement with Chaijan et al. [20], who reported that MUFA and PUFA contents in 15 days ice stored sardine (*Sardinella gibbosa*) muscle decreased by 9.7% and 8.1%, respectively, whereas SFA content increased by 2.3%. Šimat et al. [22] also reported that PUFA in farm-affected wild bogues (*Boops boops,* Linnaeus, 1758) was reduced over a storage period of 16 days in ice. 

### 3.2. Heme Iron Content

Heme iron is an important catalyst of lipid oxidation. The heme iron content in muscle was significantly decreased in fish stored in ice for an extended period and in the corresponding washed mince (Table 1). This might be due to the breakdown of the heme iron complex, induced by oxidative cleavage of the porphyrin ring during ice storage. Thiansilakul et al. [23] also reported that the heme iron content of seabass (*Lates calcarifer*) and red tilapia (*Oreochromis mossambicus* and *O. iloticus*) muscles decreased after 15 days in ice storage. The authors suggested that the disruption of heme protein and release of heme iron occurred during ice storage. Rezaei and Hosseini [24] found that heme content in whole rainbow trout (*Oncorhynchus mykiss*) also decreased with 20 days of ice storage, which was due to the release of free iron from heme. The heme iron content in washed mince was lower than that of unwashed mince (Table 1). The washing process removed water-soluble heme proteins, leading to less heme iron in the washed mince.

### 3.3. TBARS Value

The TBARS value of the different fish tissues increased with ice storage time (*p* < 0.05, Table 1). The belly flap was found to have the highest TBARS value, as it contained the highest amount of total lipid (*p* < 0.05, Table 1) and polyunsaturated fatty acids (Table 2). Moreover, various enzymes, including lipoxygenase, peroxidase and microsomal enzymes in viscera [25], could potentially promote lipid oxidation in the belly flap. Although heme iron content in unwashed mince was higher than that in washed mince, the TBARS value was slightly lower (Table 1). Washed mince contained higher phospholipids content with a higher proportion of PUFA, and thus it tended to be more susceptible to lipid oxidation despite the lower amount of the catalyst, heme iron. Addeen et al. [26] reported that a higher TBARS in washed chicken mince was plausibly due to the presence of membrane lipids and the removal of natural antioxidants in muscle, such as carnosine, anserine, glutathione, and polyamines, which are water-soluble compounds. Hoke et al. [27] also reported that the TBARS of washed mince increased during the first three months of frozen storage. In surimi and/or washed mince processing, lipid oxidation has not been as well studied as protein denaturation. However, our results suggested that lipid oxidation of washed mince produced from fish stored in ice for an extend period occurred to a greater extent than that produced from fresh fish. In addition, washed mince is prone to lipid oxidation, as it contains a higher proportion of membrane lipids and PUFA. 

### 3.4. Volatile Compounds

Volatile compounds, including alcohols, aldehydes and ketones, increased in the different tissues of silver carp during ice storage (Table 3). Several volatile compounds that are markers of lipid oxidation were prevalent in fish belly. These included alcohols (1-pentanol, 1-hexanol, 1-octen-3-ol, 1, -heptanol, 1-octanol) and aldehydes (hexanal, octanal, nonanal, 2-octenal), which are degradation products of peroxides. 1-Pentanol and 1-octen-3-ol are derived from the oxidation of linoleic acid. Iglesias et al. [28] reported that 1-octen-3-ol is an important volatile contributing to off-flavor, due to its low odor threshold. 1-Heptanol, 1-octanol, 1-hexanol, nonanal and (E)-2-octenal are likely degradation products of oleic acid [29]. 2,3-Octanedione is derived from the lipid oxidation of ω-6 fatty acids [30]. Hexanal is a secondary product from the oxidation of linoleic acid, typically used as a lipid oxidation marker in fish [5]. On day 14, hexanol and 1-octen-3-ol were detected at their highest level in the fish belly (Table 3). This is related to the oxidation of fatty acids, oleic acid and linoleic acid, respectively, which are abundant in the belly flap (Table 2). 1-Hexanol and 1-octen-3-ol could be used as lipid oxidation markers of belly flap of silver carp.

In skin, 1-hexanol, 1-octen-3-ol, 1-octanol and nonanal increased during the ice storage of slver carp. These compounds are derived from oxidation of oleic acid and linoleic acid, which were also found to be abundant in silver carp skin (Table 3). After 14 days of storage, 1-octen-3-ol and nonanal were predominant volatile compounds, and nonanal was found to be the highest in all tissues. Therefore, 1-octen-3-ol and nonanal could be considered lipid oxidation markers of silver carp skin.

1-Hexanol, 1-octen-3-ol, nonanal and 2,3-octanedione were found to increase in mince during ice storage (Table 3). 1-Hexanol was likely to be derived from the degradation products of oleic acid, which was the most abundant fatty acid in mince (Table 2). Several ketones have been identified in dry-cured fish and regarded as a sign of fish spoilage [31]. Lower levels of volatile compound were detected in washed mince samples. This indicated that washing can efficiently remove volatile compounds that cause off-odor. Only low levels of hexanal were detected in washed mince prepared from aged fish (14 days ice storage). Hexanal have also been found to be a predominant aldehyde in commercial silver carp surimi [32]. Differences in volatile compounds from the different tissues of silver carp are mainly due to variations in lipid content and fatty acid composition. The belly flap of silver carp is susceptible to lipid oxidation, generating volatile compounds, especially 1-hexanol and 1-octene-3-ol and hexanal, which likely contribute to off-odor in fish stored in ice for an extended period.

### 3.5. FT—Raman Spectroscopy

Changes in the selected FT-Raman wavenumbers of extracted lipid from the different tissues of silver carp during ice storage and in its corresponding washed mince are shown in Table 4. A decrease in the Raman band at 3015 cm^−1^ was observed in spectra from belly flap and unwashed and washed mince during ice storage. In addition, a decrease in the Raman band at 1267 cm^−1^ was observed from the analysis of the belly flap and mince during ice storage of raw material. A decrease in the two Raman bands at 3015 and 1267 cm^−1^ corresponds to *cis* =CH stretching and =CH symmetric rock *cis* double bond vibration, respectively, which indicated a reduction in unsaturated fatty acids during ice storage. Our results suggested that the Raman intensity of the cis-olefinic group =C-H stretching vibration at the 3015 cm^−1^ band and the =CH symmetric rock cis double bond at 1267 cm^−1^ can be used to monitor the oxidation of the extracted lipids. A decrease in the Raman bands of methylene groups (2935 cm^−1^ CH_2_ asymmetric, 2850 cm^−1^ symmetric stretching, 1438 cm^−1^ the CH_2_ deformation and 1301 cm^−1^ CH_2_ in-phase twisting) was also observed in lipids extracted from washed mince (Table 4, *p* < 0.05). An increase in the band at 1747 cm^−1^ was found in all samples during ice storage, corresponding to the ν(C=O) stretching of peroxides. Thus, the Raman band at 1747 cm^−1^ can be used to monitor the progress of the oxidation of lipids extracted from different tissues of silver carp. Another strong band at 1658 cm^−1^ corresponding to the *cis* double bond (C=C) stretching motion appeared to decrease in the spectra of the belly flap and mince during ice storage of raw material. This was likely due to the loss of conjugated double bonds, which was concomitant with a decrease in MUFA and PUFA (Table 2). Our results are in agreement with Chen et al. [8] who found that the Raman band at 1655 cm^−1^, assigned to ν(C=C), decreased after repeated freeze-thaw of beef. They suggested that the oxidation reduced the total unsaturation of lipid, resulting in a decrease in the C=C band. Our study demonstrates that Raman spectroscopy can be potentially used to follow the progress of lipid oxidation of silver carp during ice storage, as well as that of the respective washed mince.

### 3.6. FTIR

Changes in the distinct FTIR bands of lipid extracted from the different tissues of silver carp during ice storage and those extracted from washed mince prepared from fish at various ice storage time are shown in Table 5. A decrease in the peak areas at 3013 cm^−1^, corresponding to cis =C-H stretching, was observed in all samples (*p* < 0.05). The band observed at 3012 cm^−1^ is related to the stretching vibration of cis olefinic =C–H double bonds [33,34]. A continuous decrease in this band with extended storage time indicated the loss of *cis* double bonds. This also corresponded to changes in the Raman band at 3015 cm^−1^ (Table 4). Volpe et al. [35] reported an increase in the FTIR band at 3011 cm^−1^ in trout fillets stored at 4 °C for up to 12 days. It should be noted that the changes in FTIR bands at 3011–3015 cm^−1^ of extracted lipid in this study had trends the differed from those detected in fish flesh in situ [35]. Fish flesh is composed of other components, including proteins, glycogen and nucleotides, which could interact to some extent with lipids and/or peroxides during storage. On the other hands, extracted lipid fractions contain only lipids, fatty acids and some degradation products of lipid oxidation. FTIR measurement of the extracted lipids can better reflect the extent of lipid oxidation than measuring samples from the flesh of whole fish. Changes in methylene groups at 2922 and 2853 cm^−1^, representing asymmetric and symmetric stretching vibrations of methylene (−CH_2_) and methyl (−CH_3_) group, respectively [33,36], were rather subtle. In addition, the FTIR band at 721 cm^−1^, representing the bending vibrations of –(CH_2_) n–, HC=CH– (cis) groups [33,37], were comparable in all samples. The band at 722 cm^−1^ was also assigned to the out-of-plane bending of a cis-disubstituted group. Lipids extracted from silver carp stored for an extended period tended to produce lower values at 722 cm^−1^, suggesting the loss of cis double bonds and isomerization to a trans configuration, which commonly occurred in lipid oxidation. Changes observed in all methylene groups (2924, 2853, 722 cm^−1^) reflect structural changes in fatty acids induced by lipid oxidation, particularly lipids extracted from washed mince, which showed a decrease in Ʃ methylene groups as storage time of raw material in ice increased (*p* < 0.05, Table 5). Our results indicated that silver carp stored in ice for an extended period of time exhibited a higher degree of lipid oxidation. When these raw materials were used to prepare washed mince, the resulting product contained higher oxidized lipids content, even after extensive washing (3 cycles). These changes can be monitored using either FTIR or FT-Raman spectroscopy, which is a rapid technique that requires fewer chemicals when compared with the classical peroxide/TBARS analysis. Carbonyl compounds are indicated by the wavenumber at 1744 cm^−1^, which is related to the stretching vibration of triglyceride ester carbonyl (C=O) [37,38]. An increase in the band intensity at 1745 cm^−1^ was observed in all samples during ice storage (*p* < 0.05, Table 5). Volpe et al. [35] reported that the FTIR band at 1743 cm^−1^ was associated with peroxidation of fatty acids, which increased over time during the storage of trout fillets. Thus, an increase in the FTIR band intensity at 1745 cm^−1^ implied the formation of peroxides and/or secondary oxidation products. This was concomitant with an increase in TBARS value during the ice storage of all samples (Table 1).

An increase in the IR band 970 cm^−1^, implying an increase in −HC=CH-isolated trans double bonds, was observed in lipids extracted from belly flap of 14 days iced fish. In addition, lipid extracted from washed mince had higher level of trans fat than those extracted from unwashed mince (*p* < 0.05), suggesting a greater extent of lipid oxidation in the former. The higher content of phospholipids in washed mince and the washing process, in which agitation with the incorporation of air is continually applied, could be important factors that contribute to higher lipid oxidation of washed mince.

In the skin and belly, the phospholipids observed at 925 cm^−1^ and 825 cm^−1^, corresponding to P-O-C symmetric and asymmetric stretching, respectively, appeared to undergo subtle changes with increasing storage time (*p* > 0.05, Table 5). However, these IR bands decreased in mince stored in ice for an extended period and in the respective washed minces. These FT-IR results are well correlated with phospholipid content analyzed using the colorimetric method (Table 1). This implied that oxidation of phospholipid induced by autooxidation and/or by the action of phospholipase took place during the ice storage of fish. It should be mentioned that phosphate groups were not detected in FT-Raman spectra. Therefore, the FT-IR and FT-Raman techniques can complement each other to reveal information about both polar and nonpolar moieties in lipids.

### 3.7. Principal Component Analysis (PCA)

**Figure 1:**
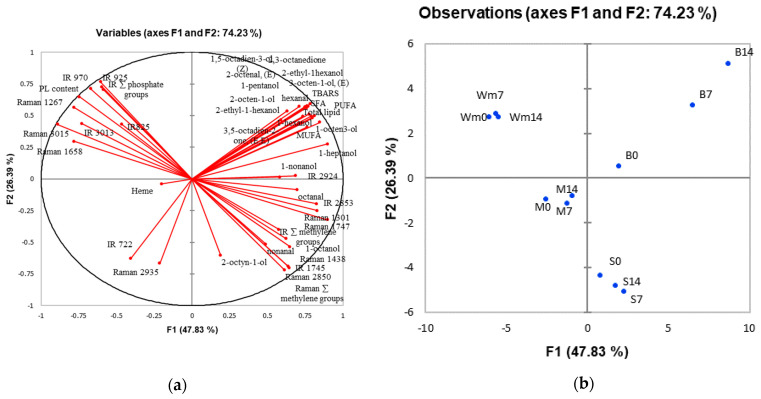
Correlation loading plot (**a**) and score plot (**b**) of principal component analysis describing measured quality parameters, namely volatile compounds, fatty acid profiles, lipid oxidation indicators and band intensity obtained from FT-IR and FT-Raman spectroscopy of skin (S), belly flap (B), and mince (M) of silver carp stored in ice for various times 0, 7 and 14 days and washed mince (Wm) prepared from fish stored in ice for 0, 7, and 14 days.

The first two components of PCA explained 74.23% of the variation (Figure 1). The skin, belly flap and unwashed and washed mince are clearly separated in different quadrants (Figure 1). The belly flap was characterized by high levels of lipid, TBARS and volatile compounds, particularly 1-octen-3-ol and 1-heptanol, which increased with storage time. Oil extracted from the belly flap was characterized by a Raman band at 1747 cm^−1^, the summation of methylene group detected by Raman spectroscopy (Raman **∑**methylene), and an IR band at 1745 cm^−1^. The intensity of these spectra increased with the storage time of the raw material. Thus, these bands could be used as markers of lipid oxidation in the belly flap, along with 1-octen-3-ol and 1-heptanol as volatile markers. Mince samples are located in the PCA quadrant opposite to the belly flap, indicating lower contents of fat and volatile compounds. It should be noted that the effect of storage time on the measured parameters is not as well correlated for mince as it is for the belly flap. Our study suggests that the belly flap should be a target tissue for monitoring the extent of the lipid oxidation of silver carp during ice storage. For washed mince sample, phospholipid content is a distinct characteristic, while IR bands that indicate phosphate group (925, 825 cm^−1^) are notable in the oil extracted from washed mince (Figure 1). This could be a potential marker to indicate the degree of lipid oxidation in the washed mince, which correlated with the freshness quality of raw material stored in ice.

## 4. Conclusions

Lipid oxidation of silver carp varied among the different body parts of fish. The belly flap of silver carp was the most susceptible part to lipid oxidation during prolonged ice storage. Fatty acids in all parts decreased as the ice storage time was extended. 1-Hexanol and 1-octen-3-ol were key volatile compounds detected in the belly of silver carp, and they increased with storage time. Washing can efficiently remove volatile compounds that cause off-odor. FTIR and FT-Raman spectroscopy revealed changes of *cis* double bonds, methylene groups, phosphate groups and ester bonds and the formation of trans isomerized fatty acids of lipids extracted from different parts of silver carp at various storage time. The Raman band at 1747 cm^−1^ could serve as a potential marker to indicate the extent of the lipid oxidation of oil extracted from the belly. The IR band at 925, 825 cm^−1^ could be used to monitor the extent of the lipid oxidation of washed mince, which is well correlated with the freshness quality of the raw material. To maintain the quality of silver carp mince and its respective washed mince, the fish belly should be removed before ice storage and before the mechanical deboning process.

## Figures and Tables

**Table 1:** Chemical composition changes of silver carp during iced storage for 14 days and their respective thrice-washed mince (dry basis).

Storage Time (Days)	Skin			Belly			Mince			Washed Mince		
	0	7	14	0	7	14	0	7	14	0	7	14
**Total lipid** **(g**/**100 g)**	8.78 ± 0.34 ^a^	8.12 ± 0.55 ^a^	7.07 ± 0.60 ^b^	40.52 ± 1.86 ^a^	38.60 ± 1.98 ^a^	33.86 ± 2.74 ^b^	11.45 ± 0.15 ^a^	10.74 ± 0.33 ^b^	9.00 ± 0.14 ^c^	6.40 ± 0.52	6.29 ± 0.85	5.83 ± 0.56
**Phospholipid** **(g PC/100 g)**	0.52 ± 0.08	0.51 ± 0.09	0.47 ± 0.12	1.15 ± 0.18	1.10 ± 0.21	0.99 ± 0.22	1.67 ± 0.27 ^a^	1.38 ± 0.17 ^b^	1.19 ± 0.20 ^b^	2.49 ± 0.23	2.35 ± 0.41	2.17 ± 0.36
**Thiobarbituric acid reactive substances (TBARS)** **(ng of malondialdehyde (MDA)/kg)**	0.69 ± 0.05 ^b^	0.83 ± 0.11 ^ab^	0.94 ± 0.06 ^a^	2.08 ± 0.10 ^c^	10.12 ± 0.90 ^b^	12.51 ± 0.21 ^a^	0.59 ± 0.03 ^c^	0.70 ± 0.02 ^b^	0.86 ± 0.06 ^a^	0.77 ± 0.08 ^b^	0.96 ± 0.05 ^a^	1.06 ± 0.05 ^a^
**Heme iron** **(mg/100 g)**	0.092 ± 0.01	0.090 ± 0.01	0.071 ± 0.01	0.176 ± 0.02	0.155 ± 0.04	0.156 ± 0.05	0.772 ± 0.08 ^a^	0.590 ± 0.03 ^b^	0.294 ± 0.05 ^c^	0.193 ± 0.04 ^a^	0.118 ± 0.03 ^b^	0.102 ± 0.02 ^b^

^a,b,c^ Different letters within each tissue are significantly different (*p* < 0.05). *n* = 3.

**Table 2:** Fatty acid contents of various tissues of silver carp during ice storage and their respective washed mince (mg/g dry sample).

Fatty Acids	Skin			Belly			Mince			Washed Mince		
	D0	D7	D14	D0	D7	D14	D0	D7	D14	D0	D7	D14
C10:0	0.20	0.25	0.27	1.15	1.21	1.17	0.15	0.14	0.34	0.10	0.09	0.09
C12:0	0.08	0.10	0.08	0.50	0.52	0.60	0.05	0.03	0.08	0.03	0.03	0.04
C13:0	0.04	0.07	0.05	0.32	0.39	0.43	0.03	0.04	0.06	0.00	0.02	0.03
C14:0	1.30	2.06	1.59	9.55	11.28	12.08	0.92	1.17	2.04	0.56	0.65	1.20
C15:0	0.29	0.39	0.29	0.88	0.87	0.93	0.37	0.32	0.74	0.23	0.28	0.44
C16:0	8.16	8.53	10.90	58.59	63.75	65.59	7.87	8.37	10.63	6.00	6.33	10.51
C18:0	1.49	2.20	1.90	10.74	11.71	11.94	1.86	1.61	3.36	1.18	1.19	1.31
C20:0	0.14	0.28	0.15	0.89	0.90	1.09	0.11	0.11	0.23	0.08	0.08	0.16
C21:0	0.02	0.02	0.04	0.18	0.25	0.31	0.00	0.02	0.10	0.00	0.02	0.03
C22:0	0.00	0.04	0.00	0.08	0.05	0.05	0.00	0.03	0.12	0.00	0.02	0.03
C23:0	0.00	0.00	0.03	0.07	0.20	0.22	0.00	0.00	0.00	0.00	0.00	0.00
C24:0	0.86	0.95	0.90	4.26	2.52	2.52	2.43	2.30	2.43	2.03	1.82	2.29
C14:1	0.64	0.73	0.38	3.66	4.30	3.35	0.21	0.14	0.03	0.03	0.02	0.02
C16:1	3.77	3.33	3.43	24.09	24.56	23.19	2.49	2.13	2.16	1.60	1.66	1.54
C17:1	0.74	0.64	0.58	3.72	3.48	4.11	0.48	0.40	0.41	0.34	0.32	0.39
C18:1n9t	0.28	0.06	0.17	1.08	1.59	1.41	0.16	0.14	0.14	0.12	0.11	0.12
C18:1n9c	15.13	14.14	10.01	85.16	70.87	67.39	7.12	6.46	6.22	4.93	4.44	3.95
C20:1	0.92	1.56	0.87	5.38	4.72	6.21	0.58	0.57	0.60	0.38	0.33	0.59
C22:1n9	0.07	0.04	0.03	0.16	0.25	0.26	0.03	0.03	0.03	0.02	0.02	0.02
C18:2n6t	0.15	0.10	0.12	1.20	1.20	1.15	0.06	0.06	0.04	0.04	0.05	0.09
C18:2n6c	2.80	1.80	2.17	13.12	16.74	15.15	1.91	1.68	1.74	1.61	1.22	1.18
C18:3n6	0.29	0.10	0.19	1.04	1.59	1.45	0.15	0.12	0.13	0.13	0.12	0.19
C18:3n3	3.80	2.38	2.92	24.74	23.65	22.61	2.18	1.90	2.02	1.57	1.45	1.04
C20:2	0.35	0.23	0.25	1.42	2.02	1.91	0.24	0.20	0.21	0.21	0.18	0.15
C20:3n6	0.81	0.38	0.58	2.38	2.71	2.52	0.75	0.63	0.59	0.65	0.54	0.54
C20:3n3	0.45	0.36	0.42	2.74	2.90	2.63	0.35	0.31	0.34	0.24	0.29	0.41
C20:4n6	1.15	1.41	0.91	5.20	5.05	5.54	1.82	1.42	1.45	1.64	1.57	1.53
C22:2	0.98	0.00	0.00	4.48	4.30	4.37	0.00	0.00	0.00	0.00	0.00	0.00
C20:5n3	1.02	0.00	0.00	6.40	5.71	5.20	0.97	0.95	0.58	1.25	1.18	1.17
C22:6n3	1.90	2.63	1.39	13.88	9.83	9.70	3.47	2.66	2.58	3.07	2.39	2.34
Saturated fatty acid (SFA)	12.58 ± 1.05 ^b^	14.89 ± 0.92 ^ab^	16.20 ± 0.94 ^a^	87.20 ± 1.89 ^b^	93.65 ± 2.25 ^ab^	96.93 ± 2.53 ^a^	13.78 ± 0.98 ^b^	14.14 ± 1.23 ^b^	20.13 ± 0.62 ^a^	10.21 ± 0.53 ^b^	10.55 ± 0.67 ^b^	16.11 ± 1.08 ^a^
Monounsaturated fatty acid (MUFA)	21.55 ± 0.93 ^a^	21.49 ± 0.85 ^a^	15.47 ± 0.84 ^b^	123.25 ± 2.91 ^a^	109.77 ± 1.58 ^b^	105.93 ± 2.82 ^b^	11.09 ± 0.31 ^a^	9.87 ± 0.57 ^ab^	9.59 ± 0.41 ^b^	7.42 ± 0.69	6.91 ± 0.76	6.62 ± 0.26
Polyunsaturated fatty acid (PUFA)	13.70 ± 0.92 ^a^	9.39 ± 0.83 ^b^	8.97 ± 0.70 ^b^	76.59 ± 1.18 ^a^	75.70 ± 1.09 ^ab^	72.23 ± 1.41 ^b^	11.91 ± 0.32 ^a^	9.94 ± 0.29 ^b^	9.69 ± 0.34 ^b^	10.41 ± 0.45 ^a^	8.99 ± 0.39 ^b^	8.64 ± 0.46 ^b^

^a,b^ Different letters within each tissue are significantly different (*p* < 0.05). *n* = 3.

**Table 3:** Relative signal intensities of volatile compounds of various part tissue of silver carp during ice storage and their respective washed mince.

RI	Compounds	Skin			Belly			Mince			Washed Mince		
		D0	D7	D14	D0	D7	D14	D0	D7	D14	D0	D7	D14
	**Alcohols**												
1256	1-pentanol	0.014 ± 0.004 ^a^	0.022 ± 0.005 ^a^	N.D.	0.057 ± 0.014 ^b^	0.259 ± 0.052 ^a^	0.190 ± 0.064 ^a^	0.019 ± 0.009	0.025 ± 0.005	0.056 ± 0.034	N.D.	N.D.	N.D.
1360	1-hexanol	0.040 ± 0.012 ^b^	0.174 ± 0.047 ^a^	0.198 ± 0.027 ^a^	0.491 ± 0.259 ^b^	1.642 ± 0.065 ^a^	1.432 ± 0.055 ^a^	0.199 ± 0.154 ^b^	0.319 ± 0.049 ^ab^	0.524 ± 0.039 ^a^	0.039 ± 0.010	0.050 ± 0.017	0.053 ± 0.017
1456	1-octen-3-ol	0.029 ± 0.017 ^c^	0.225 ± 0.004 ^b^	0.285 ± 0.012 ^a^	0.131 ± 0.059 ^b^	0.926 ± 0.119 ^a^	1.107 ± 0.520 ^a^	0.048 ± 0.006 ^b^	0.099 ± 0.013 ^ab^	0.175 ± 0.073 ^a^	0.041 ± 0.013	0.052 ± 0.028	0.048 ± 0.022
1460	1-heptanol	0.018 ± 0.001 ^b^	0.095 ± 0.031 ^a^	0.065 ± 0.015 ^ab^	0.049 ± 0.031 ^b^	0.162 ± 0.010 ^a^	0.175 ± 0.042 ^a^	0.020 ± 0.008 ^b^	0.029 ± 0.003 ^b^	0.064 ± 0.009 ^a^	0.012 ± 0.007	0.016 ± 0.002	0.018 ± 0.001
1488	1,5-octadien-3-ol, (Z)-	N.D.	N.D.	N.D.	N.D.	0.171 ± 0.022 ^a^	0.279 ± 0.139 ^a^	N.D.	N.D.	N.D.	N.D.	N.D.	N.D.
1492	2-Ethyl-1-hexanol	0.062 ± 0.015 ^ab^	0.073 ± 0.020 ^a^	0.033 ± 0.014 ^b^	0.388 ± 0.232	0.354 ± 0.0353	0.396 ± 0.068	N.D.	0.111 ± 0.049 ^a^	0.042 ± 0.035 ^b^	0.230 ± 0.039 ^a^	0.037 ± 0.015 ^b^	N.D.
1562	1-octanol	0.038 ± 0.009 ^b^	0.153 ± 0.029 ^a^	0.101 ± 0.023 ^a^	0.018 ± 0.013 ^b^	0.073 ± 0.004 ^a^	0.068 ± 0.028 ^a^	N.D.	0.028 ± 0.015 ^a^	0.021 ± 0.006 ^a^	0.007 ± 0.001 ^a^	0.003 ± 0.001 ^b^	0.006 ± 0.001 ^a^
1621	2-octenol	0.007 ± 0.002 ^b^	0.031 ± 0.002 ^a^	0.025 ± 0.011 ^a^	0.021 ± 0.004	0.048 ± 0.008	0.087 ± 0.012	N.D.	N.D.	N.D.	N.D.	N.D.	N.D.
1665	1-nonanol	0.024 ± 0.008 ^b^	0.149 ± 0.035 ^a^	0.091 ± 0.034 ^ab^	0.041 ± 0.003	0.371 ± 0.005	0.027 ± 0.011	N.D.	N.D.	N.D.	N.D.	N.D.	N.D.
1689	2-octyn-1-ol	N.D.	0.039 ± 0.003 ^a^	0.014 ± 0.002 ^b^	N.D.	N.D.	N.D.	N.D.	N.D.	N.D.	N.D.	N.D.	N.D.
1777	2-ethyl-1-hexanol	N.D.	N.D.	N.D.	N.D.	0.044 ± 0.004	0.041 ± 0.003	N.D.	N.D.	N.D.	N.D.	N.D.	N.D.
1842	3-octen-1-ol, (E)-	N.D.	N.D.	N.D.	N.D.	0.053 ± 0.010	0.064 ± 0.007	N.D.	N.D.	N.D.	N.D.	N.D.	N.D.
	**Aldehydes**												
1081	hexanal	0.019 ± 0.024	0.029 ± 0.007	0.027 ± 0.003	0.087 ± 0.034 ^b^	0.144 ± 0.054 ^b^	0.440 ± 0.135 ^a^	N.D.	N.D.	N.D.	0.014 ± 0.012 ^b^	0.033 ± 0.005 ^b^	0.055 ± 0.005 ^a^
1281	octanal	N.D.	0.070 ± 0.009 ^a^	0.026 ± 0.004 ^b^	N.D.	0.027 ± 0.001 ^b^	0.061 ± 0.018 ^a^	N.D.	N.D.	N.D.	N.D.	N.D.	N.D.
1392	nonanal	0.032 ± 0.008 ^c^	0.161 ± 0.036 ^b^	0.242 ± 0.029 ^a^	0.041 ± 0.008 ^b^	0.063 ± 0.007 ^b^	0.102 ± 0.017 ^a^	0.022 ± 0.007 ^b^	0.039 ± 0.003 ^ab^	0.064 ± 0.014 ^a^	0.013 ± 0.012	0.019 ± 0.003	0.020 ± 0.008
1430	2-octenal, (E)-	N.D.	N.D.	N.D.	N.D.	0.035 ± 0.006 ^b^	0.073 ± 0.010 ^a^	N.D.	N.D.	N.D.	N.D.	N.D.	N.D.
	**Ketones**												
1320	2,3-octanedione	N.D.	0.023 ± 0.002 ^b^	0.035 ± 0.007 ^a^	0.049 ± 0.003 ^b^	0.234 ± 0.053 ^a^	0.291 ± 0.089 ^a^	N.D.	0.037 ± 0.008 ^b^	0.142 ± 0.028 ^a^	N.D.	N.D.	N.D.
1576	3,5-octadien-2-one, (E,E)-	N.D.	N.D.	N.D.	N.D.	N.D.	0.024 ± 0.003	N.D.	N.D.	N.D.	N.D.	N.D.	N.D.

N.D. = not detected. ^a,b,c^ Different letters within each tissue are significantly different (*p* < 0.05). *n* = 3.

**Table 4:** Relative integrated intensity of selected regions of Raman spectra of lipids extracted from various tissues of silver carp during ice storage and their respective washed minces.

Wavenumber (cm^−1^)	Band Assignment	Skin			Belly			Mince			Washed Mince		
		D0	D7	D14	D0	D7	D14	D0	D7	D14	D0	D7	D14
3015	cis-olefinic group =CH stretching	7.374 ± 0.14	7.260 ± 0.11	7.279 ± 0.06	7.763 ± 0.30 ^a^	7.525 ± 0.22 ^b^	7.151 ± 0.18 ^c^	8.463 ± 0.11 ^a^	8.098 ± 0.23 ^ab^	7.964 ± 0.23 ^b^	9.187 ± 0.14 ^a^	9.044 ± 0.10 ^ab^	8.840 ± 0.10 ^b^
∑methylene groups (2935, 2850,1438, 1301)		56.450 ± 0.51	56.003 ± 0.21	55.856 ± 0.79	55.157 ± 0.45	54.306 ± 0.84	54.133 ± 0.31	54.496 ± 0.41	53.930 ± 0.53	53.546 ± 0.64	51.845 ± 0.34 ^a^	50.632 ± 0.50 ^b^	50.645 ± 0.79 ^b^
2935	ν_as_ CH_2_	9.805	9.827	9.783	9.545	9.510	9.401	9.538	9.543	9.422	9.753	9.579	9.560
2850	ν_s_ CH_2_	32.890	32.591	32.487	31.604	31.140	31.398	31.717	31.504	31.306	29.675	28.614	28.648
1438	δ(CH_2_)	9.380	9.227	9.229	9.461	9.073	8.931	8.856	8.666	8.676	8.468	8.501	8.504
1301	t CH2 in-phase twisting	4.375	4.358	4.357	4.547	4.583	4.403	4.384	4.217	4.143	3.949	3.938	3.933
												
1747	ν(C=O) carbonyl compounds	1.421 ± 0.01 ^b^	1.526 ± 0.07 ^ab^	1.576 ± 0.03 ^a^	1.519 ± 0.04 ^b^	1.575 ± 0.14 ^b^	1.606 ± 0.07 ^a^	1.255 ± 0.12 ^b^	1.455 ± 0.03 ^a^	1.449 ± 0.04 ^a^	0.991 ± 0.04 ^b^	1.078 ± 0.09 ^ab^	1.152 ± 0.04 ^a^
1658	ν(C=C) conjugated double bonds	11.119 ± 0.52	11.175 ± 0.60	10.901 ± 0.29	11.606 ± 0.41 ^a^	11.210 ± 0.11 ^ab^	10.739 ± 0.07 ^b^	11.790 ± 0.38 ^a^	11.287 ± 0.07 ^ab^	10.994 ± 0.12 ^b^	11.853 ± 0.22	11.874 ± 0.28	11.725 ± 0.11
1267	δ(=CH) symmetric rock (cis)	2.541 ± 0.10	2.517 ± 0.23	2.497 ± 0.19	3.503 ± 07 ^a^	3.231 ± 0.09 ^ab^	3.051 ± 0.17 ^b^	3.549 ± 0.02 ^a^	3.346 ± 0.08 ^ab^	3.072 ± 0.04 ^b^	3.895 ± 0.09	3.867 ± 0.07	3.701 ± 0.07

Abbreviation: s, symmetric; vs, asymmetric; ν, stretch; δ, deformation; r, rock; ^a,b,c^ Different letters within the same row of each treatment are significantly different (*p* < 0.05).

**Table 5:** Relative integrated intensity of selected regions of Fournier transforms infra-red (FT-IR) spectra of extracted lipid samples from various tissues of silver carp during ice storage and their respective washed minces.

Wavenumber (cm^−1^)	Band Assignment	Skin			Belly			Mince			Washed Mince		
		D0	D7	D14	D0	D7	D14	D0	D7	D14	D0	D7	D14
3013	Olefinic ν(=C-H) (cis)	2.685 ± 0.10 ^a^	2.538 ± 0.04 ^ab^	2.508 ± 0.10 ^b^	2.980 ± 0.12 ^a^	2.743 ± 0.15 ^ab^	2.635 ± 0.09 ^b^	3.203 ± 0.12 ^a^	3.035 ± 0.35 ^a^	2.895 ± 0.31 ^a^	3.558 ± 0.20 ^a^	3.243 ± 0.17 ^ab^	2.913 ± 0.22 ^b^
∑methylene groups (2924, 2853, 722)		32.507 ± 0.57	32.057 ± 0.56	32.065 ± 0.79	32.636 ± 0.23	32.141 ± 0.24	32.201 ± 0.42	32.516 ± 0.40	32.665 ± 0.76	31.583 ± 0.90	31.453 ± 0.51 ^a^	30.818 ± 0.39 ^ab^	29.991 ± 0.96 ^b^
2924	ν_as_(CH_2_)	15.472	15.146	15.301	16.093	15.672	15.777	15.587	15.483	15.209	14.966	15.091	14.640
2853	ν_s_(CH_2_)	12.019	11.968	11.745	12.038	12.000	12.322	11.457	12.221	11.457	11.294	11.217	11.002
722	-(CH_2_)- rocking	5.017	4.943	5.020	4.504	4.469	4.101	5.472	4.961	4.917	5.193	4.510	4.349
1745	Ester ν(C=O)	27.268 ± 0.11 ^c^	27.712 ± 0.11 ^b^	28.103 ± 0.05 ^a^	23.014 ± 0.23 ^b^	25.175 ± 0.86 ^ab^	25.455 ± 0.85 ^a^	23.292 ± 1.21 ^b^	24.068 ± 1.01 ^ab^	25.685 ± 1.16 ^a^	21.069 ± 0.32 ^b^	21.687 ± 0.26 ^ab^	22.029 ± 0.11 ^a^
970	-HC=CH-(trans) isolated double bonds	0.735 ± 0.04	0.755 ± 0.07	0.728 ± 0.09	1.693 ± 0.08 ^b^	1.827 ± 0.12 ^b^	2.188 ± 0.11 ^a^	2.074 ± 0.23	2.345 ± 0.13	2.396 ± 0.21	4.105 ± 0.19	4.111 ± 0.22	4.605 ± 0.19
∑phosphate (925, 825)		1.104 ± 0.05	1.169 ± 0.08	1.128 ± 0.17	2.050 ± 0.35	1.866 ± 0.13	1.883 ± 0.23	1.598 ± 0.05 ^a^	1.453 ± 0.04 ^b^	1.458 ± 0.04 ^b^	3.815 ± 0.12 ^a^	3.203 ± 0.09 ^b^	2.962 ± 0.13 ^b^
925	ν_s_(P-O-C)	0.717	0.889	0.605	1.326	1.213	1.282	1.296	1.183	1.171	1.957	1.934	1.947
825	ν_as_(P-O-C)	0.386	0.280	1.047	0.724	0.653	0.601	0.302	0.270	0.287	1.858	1.269	1.014

Abbreviation: s, symmetric; vs, asymmetric; ν, stretch; δ, deformation; r, rock. ^a,b,c^ Different letters within each tissue are significantly different (*p* < 0.05).

## Data Availability

Data is contained within the article.
